# Increased endothelin-1 and diminished nitric oxide levels in blister fluids of patients with intermediate cold type complex regional pain syndrome type 1

**DOI:** 10.1186/1471-2474-7-91

**Published:** 2006-11-30

**Authors:** J George Groeneweg, Frank JPM Huygen, Claudia Heijmans-Antonissen, Sjoerd Niehof, Freek J Zijlstra

**Affiliations:** 1 Department of Anesthesiology, subdivision Pain Treatment Center, Erasmus MC Rotterdam, The Netherlands

## Abstract

**Background:**

In complex regional pain syndrome type 1 (CRPS1) pro-inflammatory mediators and vascular changes play an important role in the sustained development and outcome of the disease. The aim of this study was to determine the involvement of vasoactive substances endothelin-1 (ET-1) and nitric oxide (NO) during early chronic CRPS1.

**Methods:**

Included were 29 patients with CRPS 1 who were diagnosed during the acute stage of their disease and observed during follow-up visits. Disease activity and impairment were determined and artificial suction blisters were made on the CRPS1 and the contralateral extremities for measurements of IL-6, TNF-α, ET-1 and nitrate/nitrite (NOx).

**Results:**

The levels of IL-6, TNF-α and ET-1 in blister fluid in the CRPS1 extremity versus the contralateral extremity were significantly increased and correlated with each other, whereas NOx levels were decreased.

**Conclusion:**

The NOx/ET-1 ratio appears to be disturbed in the intermediate stage of CRPS, resulting in vasoconstriction and consequently in a diminished tissue blood distribution.

## 1. Background

Complex regional pain syndrome (CRPS) is a painful disorder which mainly occurs as a complication after surgery or trauma, and the main characteristics are continuous pain, marked changes in tissue blood flow and skin surface temperature, oedema and sweating, movement disorders and trophic changes of the skin [1,2]. The severity of the symptoms is disproportionate to the initial event. The diagnosis of CRPS is entirely based upon clinical criteria [3,4]. In the Netherlands, the incidence of CRPS1 is approximately 2.6% for different fractures, which results in approximately 5100 new patients yearly, of whom a substantial part will not recover completely [5-7]. The female to male ratio is approximately 3:1, with a median age of 52.7 years at onset [7]. The pathophysiology of CRPS1 is not unravelled yet, but growing evidence indicates the involvement of an exaggerated inflammatory processes. Both central and peripheral mechanisms have been proposed to play a prominent role. Central sensitization leading to exacerbation of pain is thought to result from neuroimmune activation of cells in the peripheral nervous system [5]. During the neurogenic inflammation, neuropeptides [8] and cytokines and other mediators are released [9]. This leads to a so-called 'warm dystrophy' with signs of inflammation such as redness, increased skin temperature, loss of function and pain [4,5,10].

During the chronic, disabling stage of the disease, in general the following changes occur: i) signs of extravasation, and oedema changes into atrophy, ii) regional blood flow declines, and iii) increased skin temperatures change into diminished temperatures. Permanent damage of nerve endings can lead to less endothelium-dependent vasodilation and result in a diminished regional blood distribution, the so-called 'cold dystrophy' [11,12]. Both continuous pain and signs of 'cold dystrophy' can lead to assumed irreversible disuse. The contribution of endothelial derived factors could be crucial; endothelin-1 (ET-1) is a proven potent vasoconstrictive agent that is also believed to affect hyperalgesia [13], muscle weakness and movement disorders [14], and oedema [15].

In contrast to skeletal muscle resistance vessels, ET-1 contributes to the maintenance of skin microvascular tone through both ET_A _and ET_B _receptor-mediated vasoconstriction [16]. The initially produced pro-inflammatory cytokines tumour necrosis factor alpha (TNF-α) and interleukin-6 (IL-6) also play an important role in the initial and intermediate phases of the disease [9,17]. TNF-α is known to decrease eNOS mRNA levels by increasing the rate of mRNA degradation [18,19]. In the human forearm, TNF-α promotes ET-1 production [20]. On the other hand, ET-1 also promotes TNF-α and IL-6 production in skin-derived mast cells [21], one of the cells which seem to play a prominent role in the acute phase of CRPS1 [22].

The present study aimed to investigate the involvement of the vasoactive substances ET-1 and nitric oxide (NO) in intermediate/cold type CRPS1 in relation to inflammatory mediators.

## 2. Methods

### 2.1. Subjects

For this study 29 patients (6 males, 23 females; mean age 48 ± 11.3 (SD) years) with CRPS1 in one hand according to the criteria of Bruehl [3], were included. The patients had a mean duration of their disease of 2.8 ± 1.4 (SD) years, all being in the intermediate phase between the acute inflammatory stage and the early atrophic stage.

The mean disease activity of the patients was 35 on a scale of 0–100, calculated using the impairment sum score according to Oerlemans [23], representing a low to medium disease activity.

The Medical Ethics Committee of the Erasmus MC approved the study protocol (MEC 2004-159). The research was carried out in accordance with the Declaration of Helsinki (2000) of the World Medical Association and written informed consent was obtained from all participants.

### 2.2. Blister fluid collection

Artificial blisters were induced by means of a suction method [9,17,24-27]. A 3-hole (5 mm diameter per hole) skin suction chamber was positioned on the skin of the upper extremity, on the dorsal side of the involved hand and on the flexor side of the contralateral forearm. The latter site was used in all our previous studies to reduce the inconvenience for the patient and to enable a comparison between our studies [5,9,17,22,28,29].

A vacuum of 300 mm Hg negative pressure was applied with an Atmoforte 350A aspirator pump (ATMOS Medizintechnik, Lenzkirch, Germany), which was reduced after 15 minutes to 250 mm Hg and again, 15 minutes later, reduced to 200 mm Hg. This negative pressure was maintained until blisters containing sufficient fluid had been developed, but not longer than 2.5 hours. The contents of the blisters were punctured and pooled from each side into a 1.5 ml Eppendorff conical polypropylene tube and centrifuged for 5 minutes at 1600 × g. The mean recovery of supernatants from control blisters was 144 ± 17 (± S.E.M.) μl fluid, and 106 ± 14 μl blister fluid from the CRPS1 side. All samples were stored in 1 ml conical polypropylene tubes at -80°C until analysis.

### 2.3. Measurements of cytokines

In order to determine the contribution of pro-inflammatory cytokines to the disease activity, both IL-6 and TNF-α were determined [9,17]. Blister fluid samples were diluted 1:4 in appropriate calibrator diluent assay buffer for the direct measurement of cytokines. Cytokine assays were performed following the manufacturer's protocol (Pelikine™ human ELISA compact kits for IL-6 (cat. no. M1906) and TNF-α (cat. no. M1920), Sanquin, Amsterdam, The Netherlands). The standard curve ranges and mean calculated zero signal plus 3 SD for IL-6 were 0–450 pg/ml and 0.2 pg/ml, respectively; and for TNF-α 0–1000 pg/ml and 1 pg/ml, respectively. The requested solutions were provided with the ELISA compact kits and additional tool kits (Pelikine-Tool™ set (cat. no. M1980), Sanquin, Amsterdam, The Netherlands). Following the manufacturer's step-by-step instructions, at the end of the procedure the absorbance per well was measured at 450 nm with a Medgenix ELISA reader. Sample concentrations were calculated using the appropriate standard calibration lines and the Softmax^® ^software of the reader.

### 2.4. Measurement of endothelin-1

ET-1 concentrations were determined in paired blister fluid samples which were available for 22 patients. Blister fluids were diluted 1:2 in appropriate diluent assay buffer. An ELISA-based commercial assay kit was purchased from R&D Systems (cat. no. QET00, Abingdon, UK). The QuantiGlo ET-1 chemiluminescent immunoassay is a solid phase ELISA designed to measure ET-1 levels in human fluids without extraction procedures. It contains synthetic human ET-1 and antibodies raised against the synthetic factor, which has been shown to accurately quantitate human ET-1. A microplate luminometer was used to measure the intensity of light emitted in RLUs (relative light units). The standard curve ranged from 0–1000 pg/ml. Raw data were transferred to a personal computer in which a log concentration-logit RLU equation was performed after which sample concentrations were calculated. The minimal detectable concentration was 0.16 pg/ml.

### 2.5. Measurement of nitric oxide

Sufficient remaining sample volume was available from 17 paired blister fluid samples to perform the assay procedure for the quantitative determination of nitrate and nitrite concentrations. Samples were diluted 1:1 in appropriate diluent assay buffer. A complete assay kit was obtained from R&D Systems (cat. no. DE1500, Abingdon, UK). This assay determines NO based on the enzymatic conversion of nitrate to nitrite by nitrate reductase. The reaction is followed by a colorimetric detection of nitrite as an azo dye product of the Griess reaction. At the end of procedure the chromophoric azo derivative which absorbs light at 540 nm is detected in a Medgenix ELISA reader, after which sample concentrations are calculated using the appropriate standard calibration line by means of the Softmax^® ^software of the reader. The standard curve ranges for nitrite and nitrate were respectively 0–200 and 0–100 nmol/ml and the minimal detectable concentrations were respectively 0.22 and 0.54 nmol/ml. Sample concentrations were expressed as total NO_x _in nmol/ml.

### 2.6. Statistical analysis

The Wilcoxon signed ranks test was used for comparisons between measurements in blister fluid obtained from the CRPS1 and the contralateral extremity. Correlation coefficients between different parameters were determined by the Spearman's test for untransformed data. SPSS (version 14) for Windows was used and the significance level was set at p < 0.05.

## 3. Results

### Blister fluid

Pro-inflammatory cytokines IL-6 and TNF-α and vasoactive ET-1 and NOx were determined in blister fluid obtained from the involved CRPS1 limb and the contra-lateral limb.

**Figure 1:**
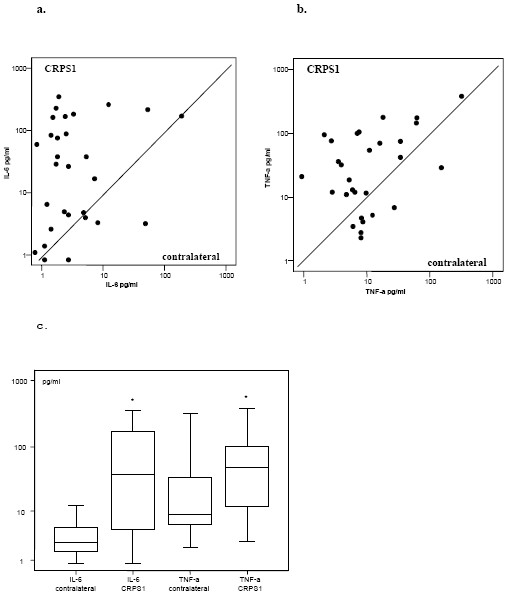
**Distribution of IL-6 and TNF-α**. Distribution of a) IL-6 data and b) TNF-α data from 29 CRPS patients, from both the involved and the contralateral extremity. In each plot, the straight line indicates per definition a CRPS to contralateral ratio of 1.00 in case no difference was observed. Values are plotted on logarithmic scales. c) Box plots of these data, indicating the median, 25–75% interval and the ranges of values, excluding outliers. The Wilcoxon signed ranks test was used to assign differences in *paired samples *(blister fluid obtained from the CRPS1 and the contralateral extremity in the same patient).

**Figure 2:**
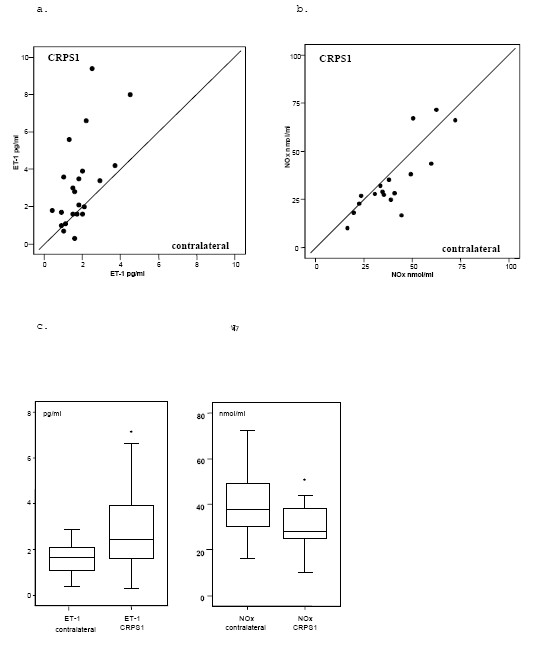
**Distribution of Endothelin 1 and Nitric Oxide**. Distribution of a) ET-1 data from 22 CRPS patients and b) NOx data from 17 CRPS patients. In each plot, the straight line indicates per definition a CRPS to contralateral ratio of 1.00 in case no difference was observed. Values are plotted on linear scales.c) Box plots of these data, indicating the median, 25–75% interval and the ranges of values, excluding outliers. The Wilcoxon signed ranks test was used to assign differences in *paired samples *(blister fluid obtained from the CRPS1 and the contralateral extremity in the same patient).

In Figures 1 and 2 the distributions of blister concentrations have been plotted as a correlation between CRPS1 samples (Y-axis) and contralateral samples (X-axis). Successively (Fig. 1a) IL-6, (Fig. 1b) TNF-α, (Fig. 2a) ET-1 and (Fig. 2b) NOx are displayed. In each plot the straight line indicates per definition a CRPS to contralateral ratio of 1.00 in case no difference was observed. Both in the IL-6, TNF-α and ET-1 distribution plots, the main number of data-points are located in the upper-left triangle, indicating a CRPS to contralateral ratio above 1.00. On the contrary, in the NOx distribution plot, the main number of data points are located in the lower-right triangle, indicating a CRPS to contralateral ratio less than 1.00.

Box plots of these data, indicating the median, 25–75% interval and the ranges of values, excluding outliers are presented in Figures 1c and 2c.

The Wilcoxon signed ranks test was used to assign differences in paired samples (blister fluid obtained from the CRPS1 and the contralateral extremity in the same patient).

Increments in the paired sample tests of IL-6, TNF-α and ET-1 were significant (p-values of 0.001, 0.003 and 0.002, respectively), whereas NOx in paired samples was significantly decreased (p-value 0.044).

Spearman's correlation coefficients between blister fluid parameters were determined. Significant correlations between IL-6, TNF-α and ET-1 were found (0.79, 0.44 and 0.67 for IL-6/TNF-α, IL-6/ET-1 and TNF-α/ET-1, respectively and p-values of p < 0.001, p = 0.039 and p = 0.001, respectively).

## 4. Discussion

This is the first study to investigate levels of ET-1 and NOx in blister fluids obtained from CRPS1-involved extremities. Apart from a study comparing ET-1 with disease severity in the skin of psoriatic patients [30], only Eisenberg et al. have investigated the possible contribution of ET-1 in CRPS [31]. In that study, ET-1 concentrations were determined in venous plasma from the contralateral limb of CRPS1 patients and did not differ significantly from values found in patients with other painful conditions and in healthy controls. The ET-1 levels found in controls and patients (mean 2.7 and 3.2 pg/ml) were, however, much higher than those reported in the literature [32,33]. Eisenberg et al. concluded that ET-1 in plasma can not be regarded as a laboratory marker for CRPS. The mean disease duration of their CRPS patients was 2 years, with a study population of relatively young patients (mean 30 years) and a male/female ratio of 2/1 [31]. These characteristics were not comparable with our study, in which age, duration of CRPS1 and the male/female ratio were much more comparable with demographic characteristics commonly found in the literature. In general, the main peak in the development of CRPS is seen at the age of 52 years and the male/female ratio is between 1:3 and 1:4.

Kuryliszyn-Moskal et al. found synovial ET-1 levels of 15.5 pg/ml [34] and similar serum levels [35]. Although the ET-1 levels we found in blister fluid (3.2 pg/ml) might be diminished due to interstitial dilution, we believe that interstitial levels better reflect local processes in CRPS1 than plasma measurements. This is confirmed by the finding of high ET-1 concentrations in blister fluids at wound regions of burn patients [36].

The role of NO in CRPS has not yet been investigated, although increased NO production from interferon-γ stimulated peripheral blood monocytes obtained from CRPS patients has been observed, in patients with apparently active disease [37]. We found an inverse relationship between ET-1 and NOx in blister samples. This has also been observed in vascular homeostasis where endothelium dysfunction plays a prominent role [38]. In venous plasma of patients with erectile dysfunction, significantly increased ET-1 and decreased NO levels were found [32]. In pathological situations circulating pro-inflammatory cytokines induce the expression of iNOS and ET-1 in smooth muscle cells, also downregulating endothelial eNOS expression, resulting in an elevated ET-1 production and a decrease of NO [18,39]. This was observed in our study: a significant increase of IL-6 and TNF-α, which causes a marked increase of ET-1 and a slight but significant decrease of NOx. These results confirm the findings of Munnikes et al. who studied patients with an intermediate duration of CRPS (median 20 months) and also found a significant elevation of IL-6 and TNF-α in the involved extremity compared with the uninvolved extremity [29]. That group consisted of 25 patients who had significantly improved on volume difference, AROM, VAS and McGill Pain Questionnaire, which makes their group comparable to our patients with a Impairment Sum Score [40] of 35. Thus there is increasing evidence for the role of proinflammatory cytokines in the initiation and development of CRPS. A longitudinal study is needed that follows selected CRPS patients from the acute to the chronic stage to determine the precise involvement of inflammatory cytokines in the disease activity.

Disuse during chronic CRPS is a common phenomenon. Vasoconstriction is usually followed by local reductions in blood flow [11,12]. It is unclear what the effect of disuse on the production of ET-1 and NO could be, but the plasma NOx concentration in healthy young humans significantly increases after exercise, with a significant decrease of ET-1 [41]. In healthy subjects, the physiological role of ET-1 in nonworking muscles during exercise has been described [42]. In healthy women, the ET-1 plasma concentration increases with age, which could be significantly counteracted after exercise [43]. Animal studies have shown that endogenous ET-1 participates in the sustained development of the disease in the redistribution of tissue blood flow [44].

In a previous study we observed that treatment with anti-TNF-α initiates recovery during the inflammatory stage of CRPS1 [17]. The effect of this intervention on the release of ET-1 and NOx is still unclear. Assuming a diminished blood flow, inhibition of the NO synthase is not advisable; on the contrary, NO donors should be supplemented [45]. Besides the smooth muscle constrictive effects of ET-1, hyperalgesia and pain could also be the result of ET receptor stimulation. Therefore, specific ET_A _receptor antagonists (such as atrasentan) could provide remission [46]. In pulmonary arterial hypertension, after treatment with the ET receptor antagonist bosentan, the suppression of NO synthesis was abolished and reversed to normal values of controls [47].

During the acute stage of CRPS1 large amounts of NO will be formed through activation of the inducible NO synthetase (iNOS). In that stage, the blood distribution has been increased which causes an increase of local skin temperature. In the endothelial cell NO will be formed from L-arginine through eNOS activation. Formation of NO could also occur after receptor stimulated activation of constitutive NOS (cNOS) or activation of neuronal NOS (nNOS) in nerve endings. In all cases this will result in increased vasodilation [48]. TNF-α counteracts the activation of eNOS [19], whereas induction of iNOS in smooth muscle cells will be stimulated to generate NO [38]. In the trophic phase of CRPS1 there will be a decline in the contribution of inflammatory mediators. Consequently, in combination with disuse of the extremity, less NO could be generated, resulting in diminished basal relaxation and retarded blood distribution, after which signs of the 'cold dystrophy' will become apparent.

## 5. Conclusion

In the vascular system a clear crosstalk exists between the NO and the ET-1 system. It seems that this interrelationship also exists in CRPS. It is obvious that NO functions as a controlling mechanism against ET-1-induced vasoconstriction. The stage of the disease, the acute or inflammatory phase, or the chronic and/or trophic phase, determines the involvement of inflammatory cytokines which could promote ET-1 production, leading to vasoconstriction and consequently to a diminished tissue blood distribution. A longitudinal study following CRPS patients during the course of the disease is needed to investigate the NOx/ET-1 ratio as an indicator of vascular involvement.

## Abbreviations

CRPS Complex Regional Pain Syndrome

NO Nitric Oxide

iNOS, eNOS, inducible, endothelial,

cNOS, nNOS constitutive and neuronal Nitric Oxide Synthetase

Nox Nitrite and Nitrate

ET-1 Endothelin

ET_A _Endothelin type A

ET_B _Endothelin type B

TNF-α Tumour Necrosis Factor alpha

IL-6 Interleukin 6

ISDN Isosorbidedinitrate

mRNA messenger Ribo Nucleid Acid

RLU Relative Light Unit

RSDSA Reflex Sympathetic Dystrophy Syndrome Association

TREND Trauma Related Neuronal Dysfunction

## Competing interests

The author(s) declare that they have no competing interests.

## Authors' contributions

JGG carried out the measurements, was responsible for the coordination of the study and wrote the final version of the manuscript. FJPMH participated in the design of the study, performed the patient selection and revised the definitive article. CHA carried out the immunoassays and assisted in writing. SN participated in the measurements the statistical analysis and corrected the final document. FJZ was responsible for the design of the study, wrote the first draft of the manuscript, performed the statistical analysis and supervised the writing process. All authors read and approved the final manuscript.

## Pre-publication history

The pre-publication history for this paper can be accessed here:
